# Effects of inflammatory and anti-inflammatory environments on the macrophage mitochondrial function

**DOI:** 10.1038/s41598-020-77370-x

**Published:** 2020-11-23

**Authors:** Dong Ji, Jian-yun Yin, Dan-feng Li, Chang-tai Zhu, Jian-ping Ye, Yuan-qing Pan

**Affiliations:** 1 Central Laboratory, Shanghai Sixth People’s Hospital, Shanghai University of Medicine & Health Sciences, No. 600 Yishan Road, Shanghai, 200233 China; 2 Department of Thyroid Breast Surgery, Kunshan Affiliated Hospital of Nanjing University of Chinese Medicine, Kunshan, 215300 Jiangsu Province China; 3 Department of Medical Psychology, Guilin Medical University, Tianjin University of Technology and Education, Campbell China Network, No.1 Zhiyuan Road, Guilin, 541010 China

**Keywords:** Biological techniques, Cell biology

## Abstract

Mitochondrial response to inflammation is crucial in the metabolic adaptation to infection. This study aimed to explore the mitochondrial response under inflammatory and anti-inflammatory environments, with a focus on the tricarboxylic acid (TCA) cycle. Expression levels of key TCA cycle enzymes and the autophagy-related protein light chain 3b (LC3b) were determined in raw 264.7 cells treated with lipopolysaccharide (LPS) and metformin (Met). Additionally, reactive oxygen species (ROS) levels and mitochondrial membrane potential were assessed using flow cytometry. Moreover, 8-week-old C57BL/6J mice were intraperitoneally injected with LPS and Met to assess the mitochondrial response in vivo. Upon LPS stimulation, the expression of key TCA enzymes, including citrate synthase, α-ketoglutarate dehydrogenase, and isocitrate dehydrogenase 2, and the mitochondrial membrane potential decreased, whereas the levels of LC3b and ROS increased. However, treatment with Met inhibited the reduction of LPS-induced enzyme levels as well as the elevation of LC3b and ROS levels. In conclusion, the mitochondrial TCA cycle is affected by the inflammatory environment, and the LPS-induced effects can be reversed by Met treatment.

## Introduction

Inflammation is a reaction to external stimuli that can both prevent and cause tissue injury^1^. While macrophage-induced inflammation is crucial for the response to pathogen infection, excessive or sustained inflammation can cause tissue damage and may lead to different conditions such as diabetes, cardiovascular diseases, and tumors^2^. Thus, the study of the inflammation process in macrophages is crucial for the understanding of cell phagocytosis, cellular immunity, and molecular immunology^3^. Lipopolysaccharide (LPS) is a gram-negative bacteria-derived molecule that can induce neutrophil infiltration and inflammation^4–6^. Metformin (Met) is the first-line drug for the treatment of type 2 diabetes mellitus^7^. Other than its hypoglycemic effect, the anti-inflammatory effect of Met is well known and reported in the literature^7–10^. Thus, LPS and Met are commonly used to establish inflammatory and anti-inflammatory environments in macrophages, respectively^5,7,11^.

Mitochondria play an important role in cell energy metabolism^12^ through the tricarboxylic acid cycle (TCA), whose main product is adenosine triphosphate (ATP), the fundamental energy molecule for cells^12^. If the ATP supply is insufficient, it can damage organs and tissues with high energy demand, such as the heart, muscles, and brain, leading to serious diseases^12^. Citrate synthase (CS), α-ketoglutarate dehydrogenase (OGDH), isocitrate dehydrogenase 2 (IDH2), and malic dehydrogenase 2 (MDH2) are key enzymes in the TCA cycle. If the expression of these key enzymes is dysregulated, the cell’s function is negatively affected.

Autophagy is one of the major digestive processes in cells and plays a critical role in maintaining cell homeostasis through delivering cytoplasmic materials, such as lipids, proteins, and organelles to lysosomes, for degradation^13^. ATG8 is a core autophagy-related complex and LC3b is an ATG8 subunit^14,15^. Cells’ status can be inferred by examining autophagy proteins such as ATG8, as well as measuring ROS and mitochondrial membrane potential.

At present, most research efforts in the inflammation field are focused on characterizing cellular and humoral immunity^12,16,17^. However, how inflammatory and anti-inflammatory environments affect macrophage mitochondrial function is poorly understood. Previous studies suggested that abnormal mitochondrial function is associated with diseases such as Parkinson's disease and diabetes mellitus^18,19^. Therefore, studying the correlation between inflammation and macrophage mitochondrial function could lead to a better understanding of the link between inflammation and diseases.

This study aims to explore the energy metabolism of macrophages under either inflammatory or anti-inflammatory conditions by adding LPS and Met, respectively, using an in vitro and an in vivo model.

## Methods

### Ethics statement

The procedures for care and use of mice model was approved by the Shanghai Jiao Tong University Affiliated Sixth People's Hospital of Medicine Ethics Committee (Shanghai, China). All animal-related methods were carried out in accordance with relevant guidelines and regulations.

### Experimental reagents

Most reagents used in this study are commercially available, including LPS (055:B5) and Met (Sigma Company, USA), RAW264.7 mouse macrophages (Cell Bank of the Chinese Academy of Sciences, China), DMEM low sugar medium (Hyclone Company, USA), fetal bovine serum and penicillin/streptomycin antibiotics (Gibco Company, USA). The following antibodies were purchased from Abcam, UK: anti-CS (ab129095), anti-OGDH (ab137773), anti-IDH2 (ab131263), anti-MDH2 (ab181873), anti-GAPDH (ab181602) and anti-LC3b (ab204297). Goat-anti-mouse (A0216) secondary antibody and goat-anti-rabbit (A0208) secondary antibody were purchased from Biosharp Co., Ltd., Hefei, China. The Reactive Oxygen Species Assay Kit (50101ES01) and JC-1 Mitochondrial Membrane Potential Assay Kit (40706ES60) were purchased from Yisheng Biotechnology Co., Ltd., Shanghai, China. BCA protein concentration detection kit, RIPA Lysis buffer, and PMSF were purchased from Beyotime Co., Ltd., Shanghai, China. RNA isolater Total RNA Extraction Reagent was purchased from Vazyme Co., Ltd., Nanjing, China. Fast Quant RT kit and qPCR kit Super Real Pre Mix Plus were purchased from Tiangen Co., Ltd., Shanghai, China.

Flow cytometer analyses were performed using a BD FACS Celesta analytical flow cytometer, quantitative PCR using a Roche Light Cycler 480II.

### Macrophage culture

RAW264.7 cells were cultured in DMEM low glycemic medium supplemented with 100 IU/mL of penicillin, 100 µg/mL of streptomycin, and 10% heat-inactivated fetal bovine serum. Cells were cultured at 37 °C with 5% CO_2_. Cells were used for the experiments when they reached the logarithmic growth phase. RAW264.7 cells were inoculated on 6-well plates with a density of 5 × 10^5^ cells/mL per well and 2 mL of medium were added to each well for 24 h. Then, cells were treated as follows: LPS group, 1 μg/mL of LPS for 8 h; Met plus LPS group, 5 mmol/L^20^ of Met for 1 h, and then 1 μg/mL of LPS for 8 h. LPS was dissolved in pure dd water and Met was dissolved in pure PBS.

### Real-time PCR (qPCR)

Total RNA was extracted from RAW264.7 cells using the RNA isolater Total RNA Extraction Reagent (Vazyme Co., Ltd., Nanjing, China). RNA concentration and purity were determined by ultraviolet spectrophotometer. Then RNA samples were reverse transcribed using the Fast Quant RT kit (Tiangen Co., Ltd., Shanghai, China), following the manufacturer's protocol. qPCR was performed using the Super Real Pre Mix Plus (Tiangen Co., Ltd., Shanghai, China) with the following PCR profile: 95 °C for 15 min, followed by 40 cycles of 95 °C for 10 s, 60 °C for 20 s, and 72 °C for 30 s. Relative expression levels were measured using the 2^−ΔΔCt^ method. Primer sequences for qPCR in this study is seen Table 1.

**Table 1:** Primer sequences for qPCR.

Gene	Forward primer 5′–3′	Reverse primer 5′–3′
GAPDH	gctcaggcctctgcgccctt	atacggactgcagccctccc
IDH2	cacttcctgaacaccacaga	gcagtaggggtggacactac
MDH2	tgaagtgagaggtgtgagcc	Atcatgtctttggaaccact
IL-6	accaagaccatccaattcat	Cattcctcactgtggtcaga
IL-10	agtgtgtattgagtctgctgg	Acctcgtttgtacctctctcc
CS	gtcctctctcagcaggtc	Actatcttctgaccttggt
OGDH	gaacagaaccctatgtggct	aggagtagtttcatcttgcta

### Western blotting

Cells and lung tissues were washed twice with PBS and lysed in RIPA Lysis buffer (Beyotime Co., Ltd., Shanghai, China). Total protein concentrations were measured using a BCA Protein Assay Kit (Beyotime Co., Ltd., Shanghai, China), and an equal amount of protein (30 µg/lane) was loaded into an SDS–polyacrylamide gel for electrophoresis and then transferred onto a polyvinylidene difluoride membrane. Membranes were then blocked by incubating them in TBS containing 5% non-fat milk and 0.1% Tween-20 for 2 h at room temperature. Membranes were then incubated sequentially with primary antibodies overnight at 4 °C. The following primary antibody dilutions were used: anti-IDH2 (1:1000), anti-CS (1:1000), anti-OGDH (1:1000), anti-MDH2 (1:10,000), anti-LC3b (1:1000), and anti-GAPDH (1:1000). Membranes were then washed 3 times in 0.1% TBST and incubated with a goat anti-mouse secondary antibody and a goat anti-rabbit secondary antibody (1:2000) for 2 h at room temperature. Proteins were visualized and images captured using the ChemiQ 4600 fluorescent chemiluminescence imaging device.

### Reactive oxygen species detection

Intracellular total ROS were detected using the Reactive Oxygen Species Assay Kit (50101ES01, Yisheng Biotechnology Co., Ltd., Shanghai, China) according to the manufacturer's protocol. Briefly, following the treatment with LPS and Met, the cell culture medium was removed and dichloro-dihydro-fluorescein diacetate (DCFH-DA) was added at a final concentration of 10 µM. Then, cells were incubated in a CO_2_ incubator for 20 min at 37 °C and washed 3 times with PBS.

The relative FITC fluorescence intensity was measured by flow cytometry as a measure of the ROS levels. The FITC excitation wavelength was 488 nm and the emission wavelength was 525 nm.

### Mitochondrial membrane potential detection

Mitochondrial membrane potential was detected using the JC-1 Mitochondrial Membrane Potential Assay Kit (40706ES60, Yisheng Biotechnology Co., Ltd., Shanghai, China), according to the manufacturer's protocol. Briefly, following treatment, the cell culture medium was removed and cells were resuspended in 0.5 mL of JC-1 staining solution and then incubated at 37 °C for 20 min in a light-proof cell incubator. Then, cells were centrifuged at 4 °C for 3 min at 600 × *g*, the supernatant was discarded, cells were washed twice with the JC-1 dye buffer, and cells were resuspended. FITC and PE fluorescence reading were used as a measure of mitochondrial membrane potential. FITC fluorescence was detected at 488 nm and 530 nm by flow cytometry, while the PE fluorescence signal was excited at 530 nm and detected at 630 nm.

### Mice management

Mice were purchased from Charles River Co., Ltd., USA (animal license: No. SCXK20190001). Eight-week-old C57BL/6J male mice were divided into three groups: control, LPS group, and Met plus LPS group (six mice in each group). Mice from the LPS group were injected with 20 mg/kg LPS and sacrificed 24 h later; mice from the Met plus LPS group were injected with 250 mg/kg Met and 1 h later were injected with 20 mg/kg LPS and sacrificed 24 h later.

### Histopathological examination of mice lung tissues

Tissues were fixed in 10% formalin at room temperature and embedded in paraffin wax. Sections of 3 µm thickness were cut from the paraffin block and mounted on poly-L-lysine-coated glass slides (Matsunami Glass Ind., Ltd., Tokyo, Japan). Paraffin sections were dewaxed at 37 °C for 7 h and rehydrated using a 70–100% alcohol gradient (70%; 90%; 95%; and three times 100%), 90 min for each interval, as previously described^21^. Paraffin-embedded sections were stained with hematoxylin and eosin (H&E) to observe changes in the alveolar septum and injury and accumulation of inflammatory factors in the alveolar cavity using a light microscope (Olympus BX50, Olympus, Tokyo, Japan).

### Statistical analysis

Median between two groups was compared using the Mann–Whitney test, while the medians of multiple groups were compared using the Kruskal–Wallis test. All statistical analyses were performed using the GraphPad Prism 5.0 (USA) statistical software and a *P* < 0.05 was considered statistically significant.

## Results

### ROS are increased following inflammation

**Figure 1:**
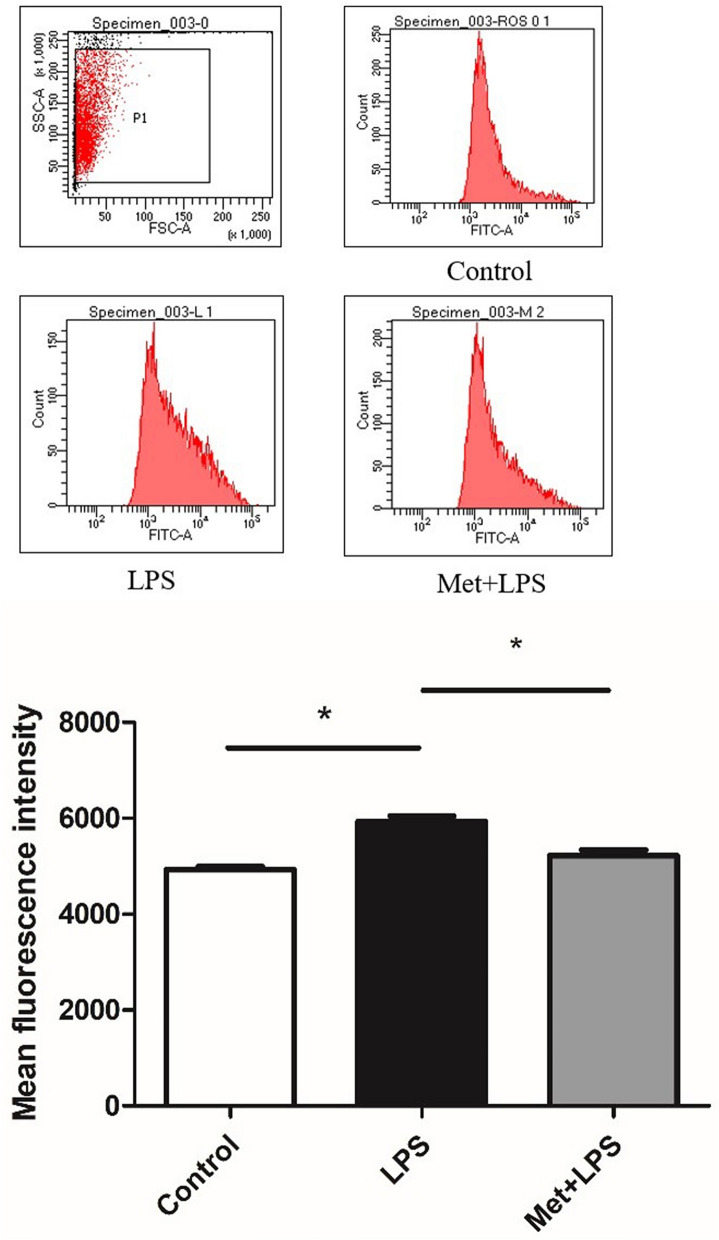
Effects of lipopolysaccharide and metformin on reactive oxygen species in macrophages. LPS: lipopolysaccharide, Met: Metformin. LPS group: compared with blank control group. Met + LPS group: compared with LPS group. *P < 0.05.

After treating RAW264.7 macrophages with LPS, we observed an increase of ROS levels, compared to the control cells (*P* < 0.05) (Fig. 1). Interestingly, this increase was inhibited when cells were pre-treated with Met (*P* < 0.05).

### LPS treatment increases the level of IL-6 and metformin increases the level of IL-10

**Figure 2:**
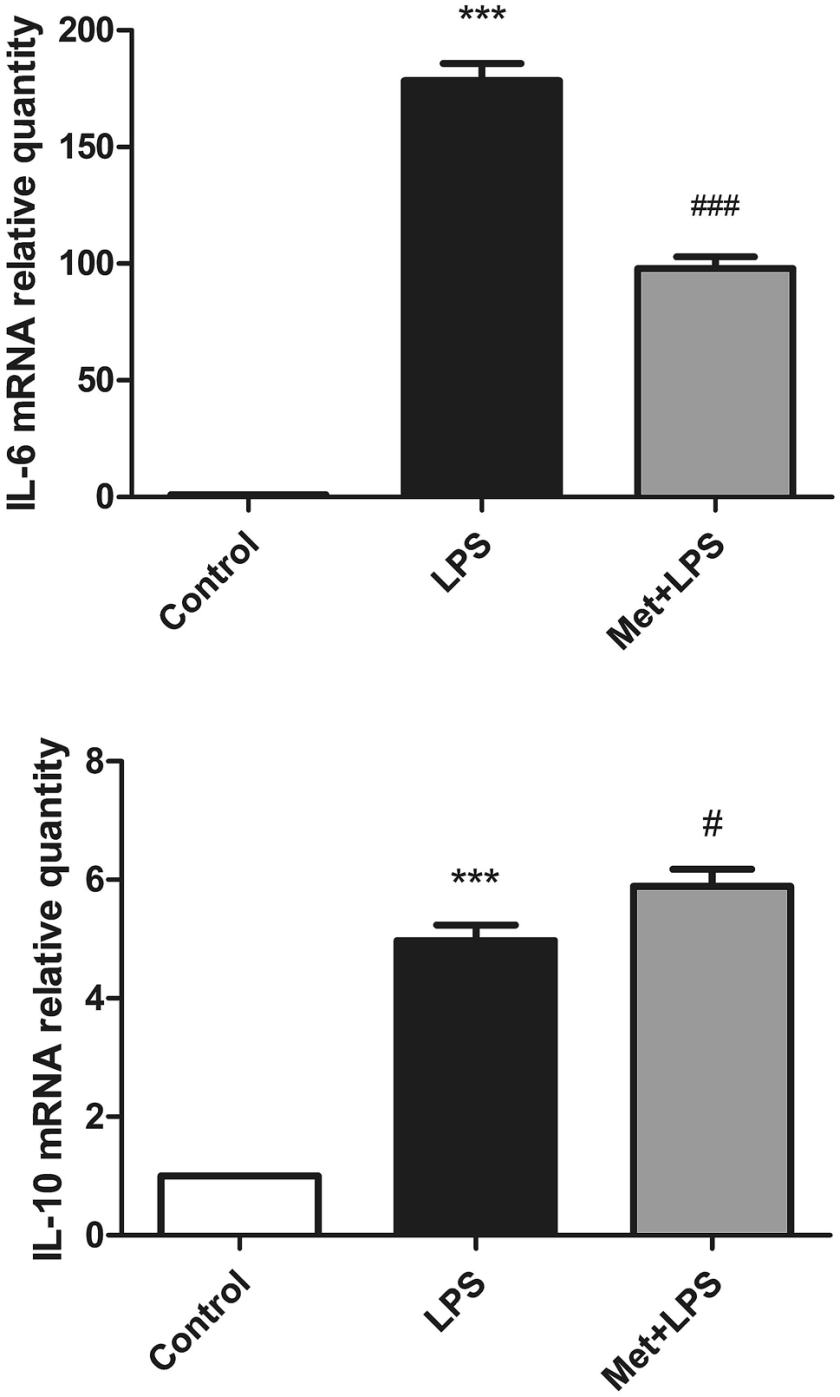
Effects of lipopolysaccharide and metformin on interleukin 6 and IL-10 in macrophages. LPS: lipopolysaccharide, Met: Metformin, IL-6: interleukin 6, IL-10: interleukin-10. *: compared with blank control group, #: compared with LPS group. *P < 0.05, **P < 0.01: ***P < 0.0001. ^#^*P* < 0.05, ^##^*P* < 0.01: ^###^*P* < 0.0001.

Similarly to ROS, we observed the increase of the pro-inflammatory marker IL-6 following LPS treatment (*P* < 0.05). Conversely, when cells where pre-treated with Met, we observed a lack of IL-6 induction (*P* < 0.05) and the concomitant increase of the anti-inflammatory marker IL-10 (*P* < 0.05) (Fig. 2).

### Expression levels of key TCA cycle enzymes decrease following inflammation

**Figure 3:**
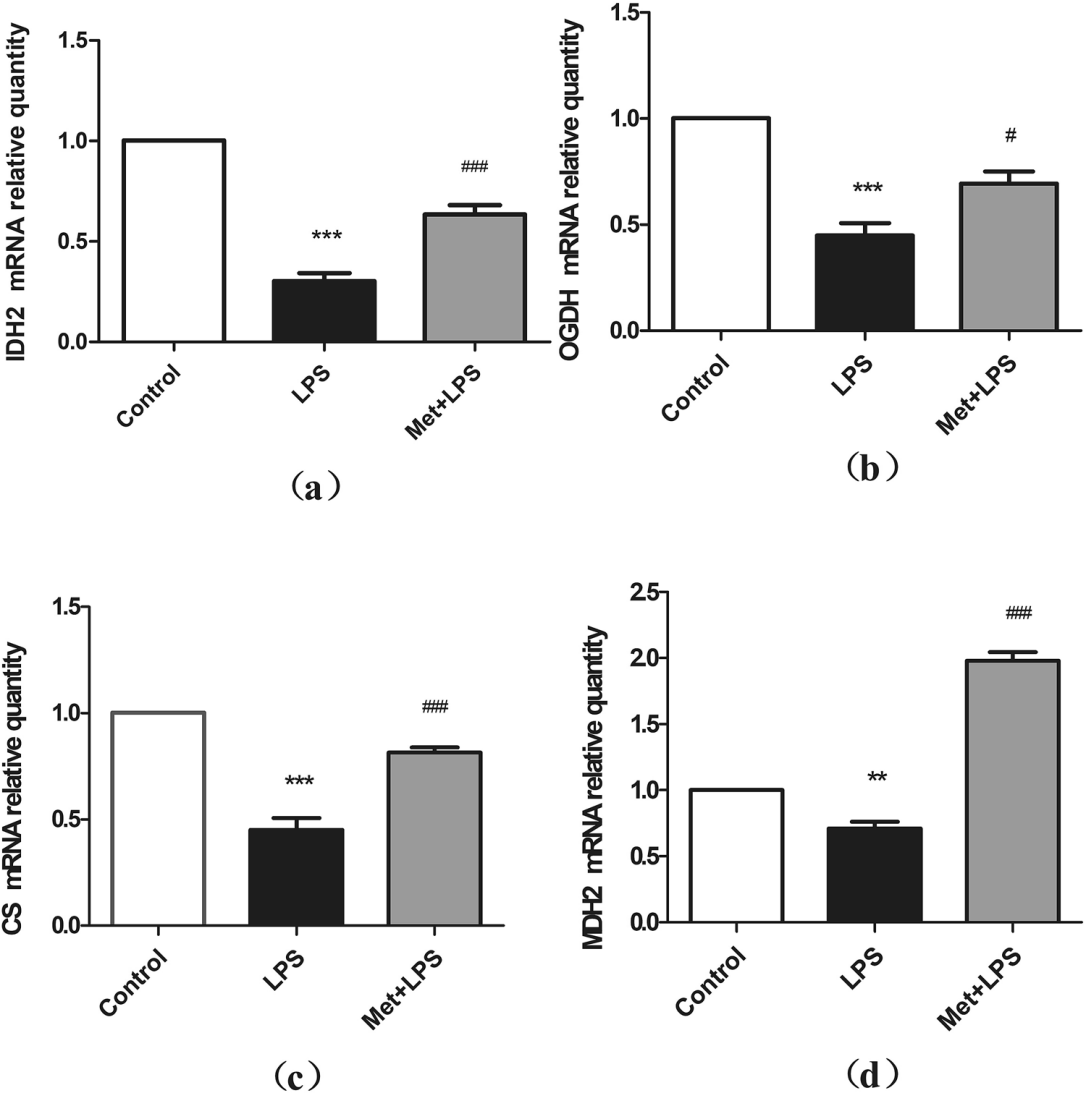
Effects of lipopolysaccharide and metformin on the mRNA expression levels of tricarboxylic acid cycle key enzymes in macrophages. (**a**) Isocitrate dehydrogenase 2 mRNA; (**b**) ɑ-ketoglutarate dehydrogenase; (**c**) Citrate synthase; (**d**) Malate dehydrogenase 2. LPS: lipopolysaccharide, Met: Metformin , CS: citrate synthase, IDH: isocitrate dehydrogenase, MDH: malic dehydrogenase, OGDH: α-ketoglutarate dehydrogenase. *: compared with blank control group, #: compared with LPS group. *P < 0.05, **P < 0.01: ***P < 0.0001. ^#^*P* < 0.05, ^##^*P* < 0.01, ^###^*P* < 0.0001.

We then measured the mRNA levels of 4 key enzymes of the TCA cycle: OGDH, IDH2, CS, and MDH2. The expression levels of all 4 genes were decreased upon LPS stimulation (*P* < 0.05), but this effect was reversed when pretreating RAW264.7 cells with Met (*P* < 0.05) (Fig. 3).

**Figure 4:**
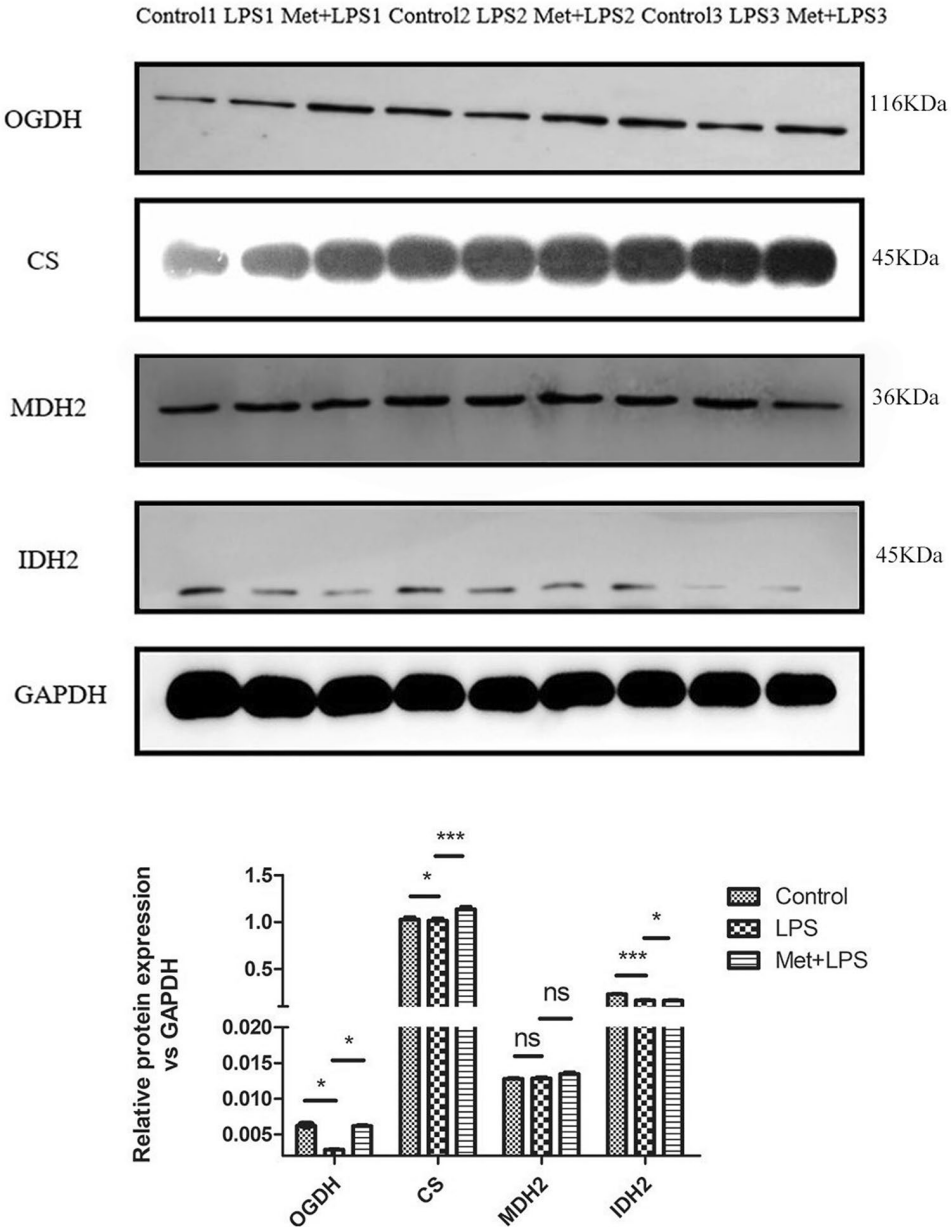
Effects of lipopolysaccharide and metformin on the protein expression levels of tricarboxylic acid cycle key enzymes in macrophages. GAPDH: glyceraldehyde-3-phosphate dehydrogenase , LPS: lipopolysaccharide, Met: Metformin , CS: citrate synthase, IDH: isocitrate dehydrogenase, MDH: malic dehydrogenase, OGDH: α-ketoglutarate dehydrogenase. LPS group: compared with blank control group. Met + LPS group: compared with LPS group. *P < 0.05, **P < 0.01, ***P < 0.0001. *ns* no significance.

We also detected the protein levels by Western blotting and observed a significant decrease of OGDH, IDH2, and CS following LPS stimulation (*P* < 0.05), which was reversed by Met pre-treatment (*P* < 0.05). However, differently from the mRNA levels, the protein levels of MDH2 did not significantly change (Fig. 4).

### Expression levels of LC3b sharply increase following inflammation

**Figure 5:**
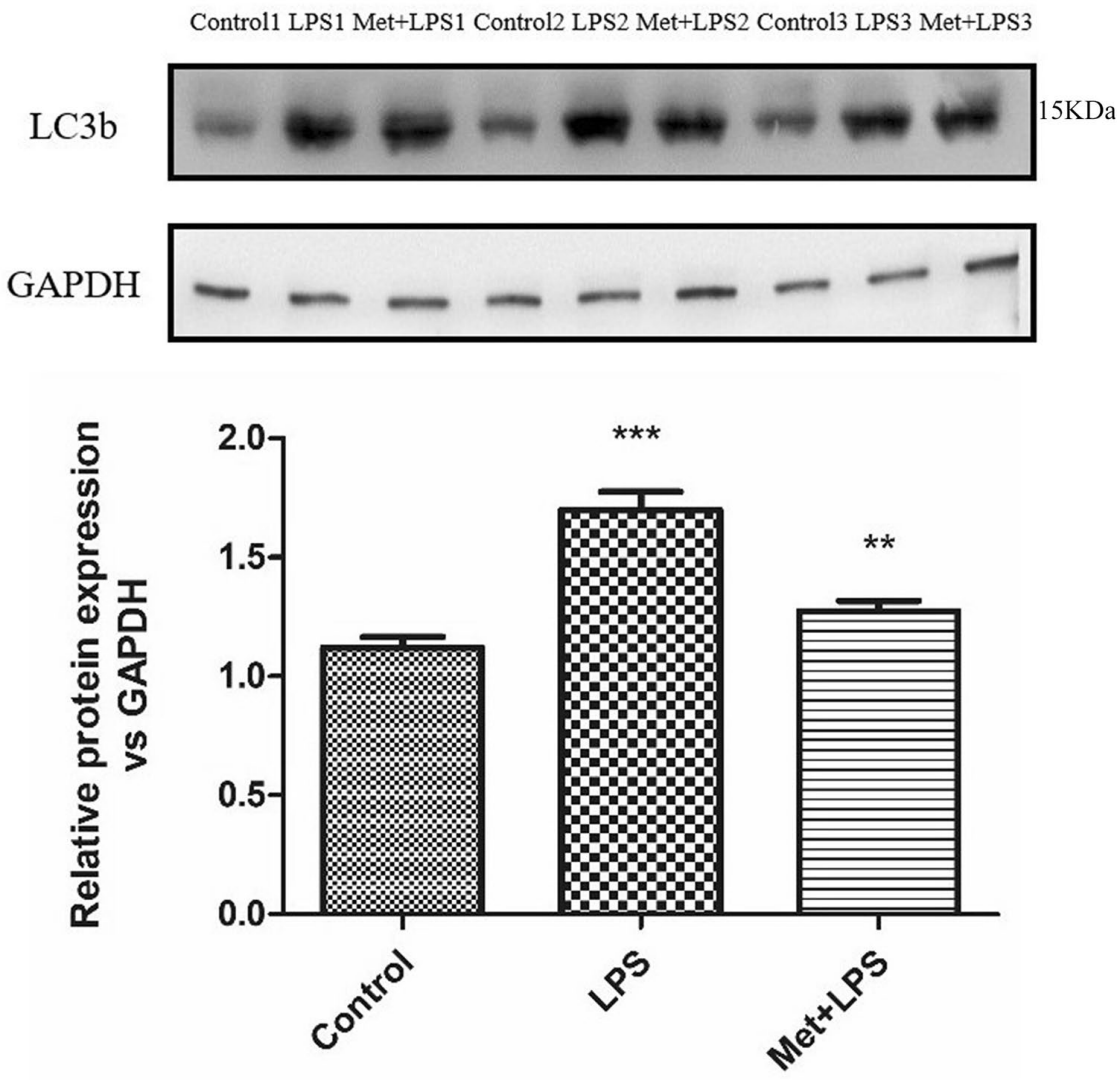
Effects of lipopolysaccharide and metformin on autophagy-related protein LC3b in macrophages. GAPDH: glyceraldehyde-3-phosphate dehydrogenase, LPS: lipopolysaccharide, Met: Metformin, LC3b: microtubule associated protein 1 light chain 3 beta. LPS group: compared with blank control group. Met + LPS group: compared with LPS group. *P < 0.05, **P < 0.01, ***P < 0.0001.

We then detected the expression of the autophagy protein LC3b in RAW264.7 macrophages treated with LPS and observed stimulant significant increase (*P* < 0.0001), which was reversed upon pre-treatment with Met (*P* < 0.01) (Fig. 5).

### Mitochondrial membrane potential decreases upon inflammation

**Figure 6:**
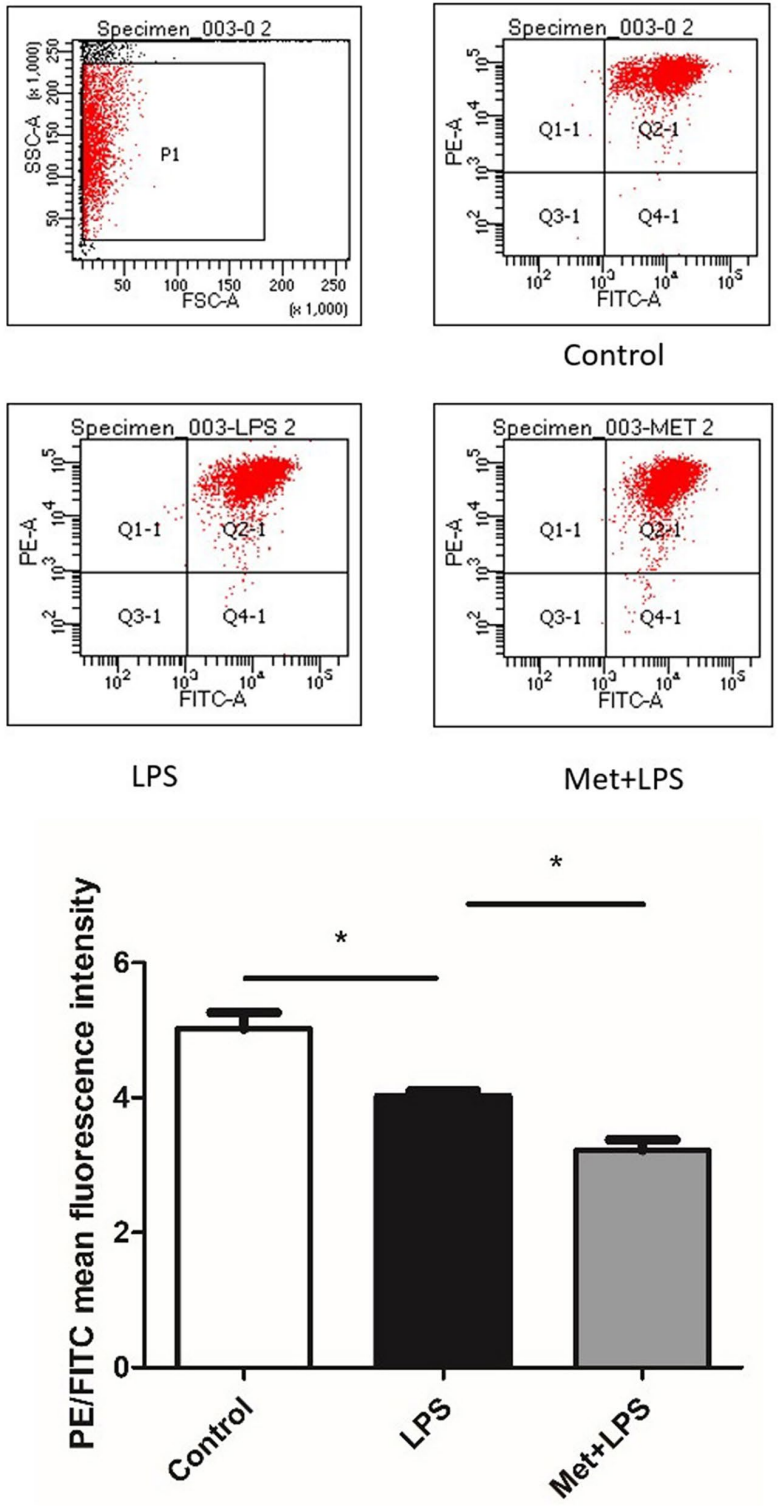
Effects of lipopolysaccharide and metformin on the membrane potential of macrophages mitochondrial. LPS: lipopolysaccharide, Met: Metformin. LPS group: compared with blank control group. Met + LPS group: compared with LPS group. *P < 0.05.

Finally, we measured the mitochondrial membrane potential in RAW264.7 macrophages treated with LPS and observed a significant decrease of the potential (*P* < 0.05), which was reversed upon pre-treatment with Met (*P* < 0.05), (Fig. 6).

### Immunohistochemical examination of lung tissues following LPS and Met/LPS treatment

**Figure 7:**
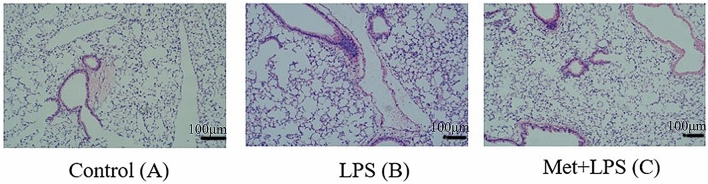
Lung histopathological examination of mice treated with lipopolysaccharide and metformin. (**A**) Normal control mice (× 100). (**B**) Septic mice (20 mg/kg of LPS for 24 h, × 100). (**C**) Septic mice with metformin (× 100). Metformin (250 mg/kg, dissolved in normal saline) was administered intraperitoneally at 1 h before LPS exposure.

We then investigated the effect of LPS and Met treatment in vivo. We observed that the injection of mice with 20 mg/kg of LPS induced the following pathological changes in the lung tissue 24 h later: significant enlargement of the alveolar septum, injury and rupture of the alveolar wall and capillary blockage, accumulation of erythrocyte in the interstitial fluid, secretion of a large number of inflammatory factors in the alveolar cavity, and infiltration of inflammatory neutrophils in the interstitium. All these parameters improved when mice where pre-treated for 1 h with 250 mg/kg Met (Fig. 7).

### Expression levels of key TCA cycle enzymes decreases following inflammation in the lung tissue

**Figure 8:**
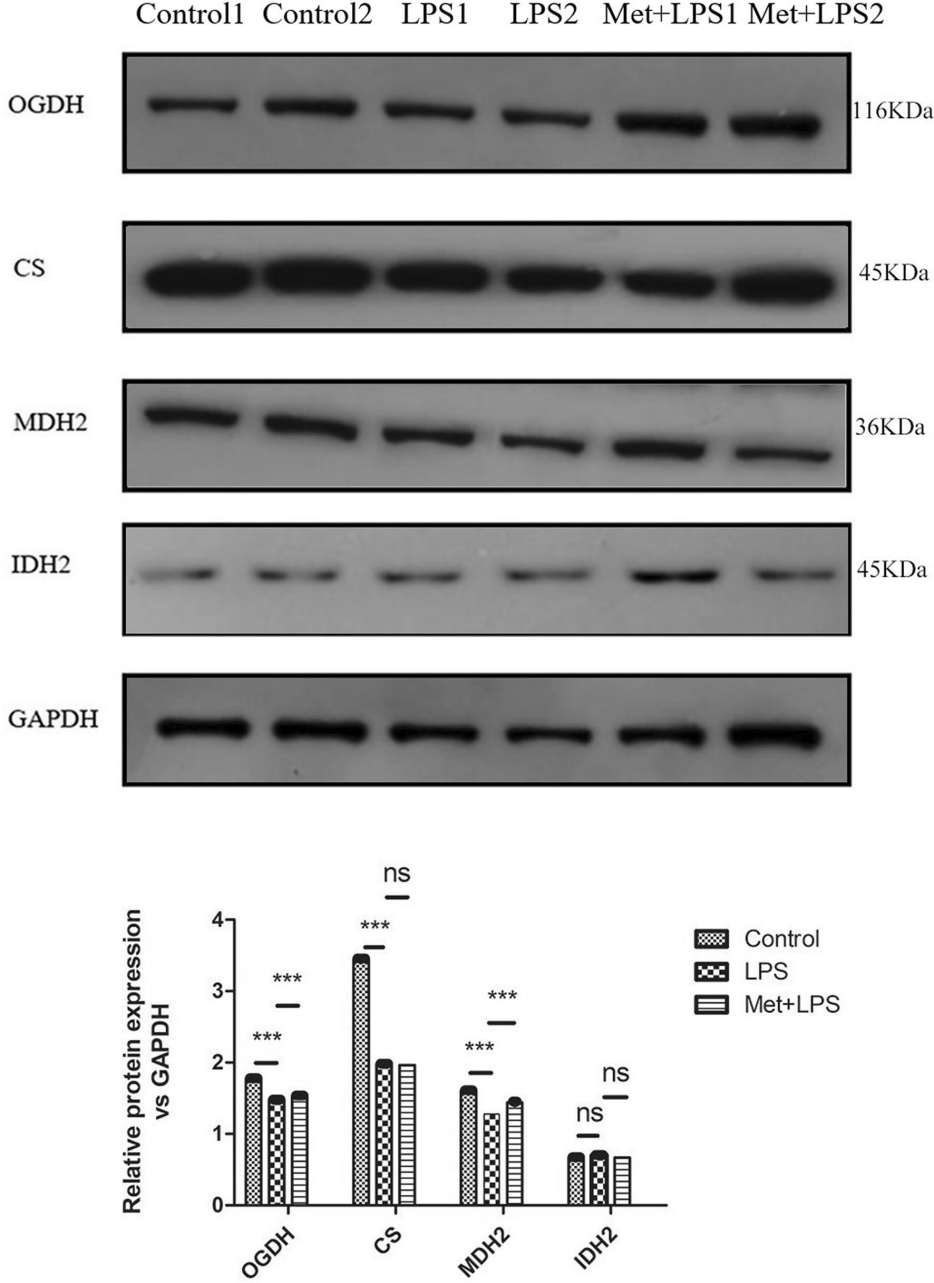
The protein expression levels of tricarboxylic acid cycle key enzymes in macrophages in mice treated with lipopolysaccharide and metformin. GAPDH: glyceraldehyde-3-phosphate dehydrogenase, LPS: lipopolysaccharide, Met: Metformin, CS: citrate synthase, IDH2: isocitrate dehydrogenase 2, MDH2: malic dehydrogenase 2, OGDH: α-ketoglutarate dehydrogenase. LPS group: compared with blank control group. Met + LPS group: compared with LPS group. *P < 0.05, **P < 0.01: ***P < 0.0001. *ns* no significance.

We observed that, upon LPS treatment, the expression of OGDH, CS, and MDH2 in the lung tissue significantly decreased (*P* < 0.05), while the expression of IDH2 did not significantly change. However, upon Met pre-treatment, the expression levels of OGDH and MDH2 were reverted (*P* < 0.05), while the expression of IDH2 and CS was not impacted (Fig. 8).

### Expression levels of LC3b increase two-fold in the lung tissue following inflammation

**Figure 9:**
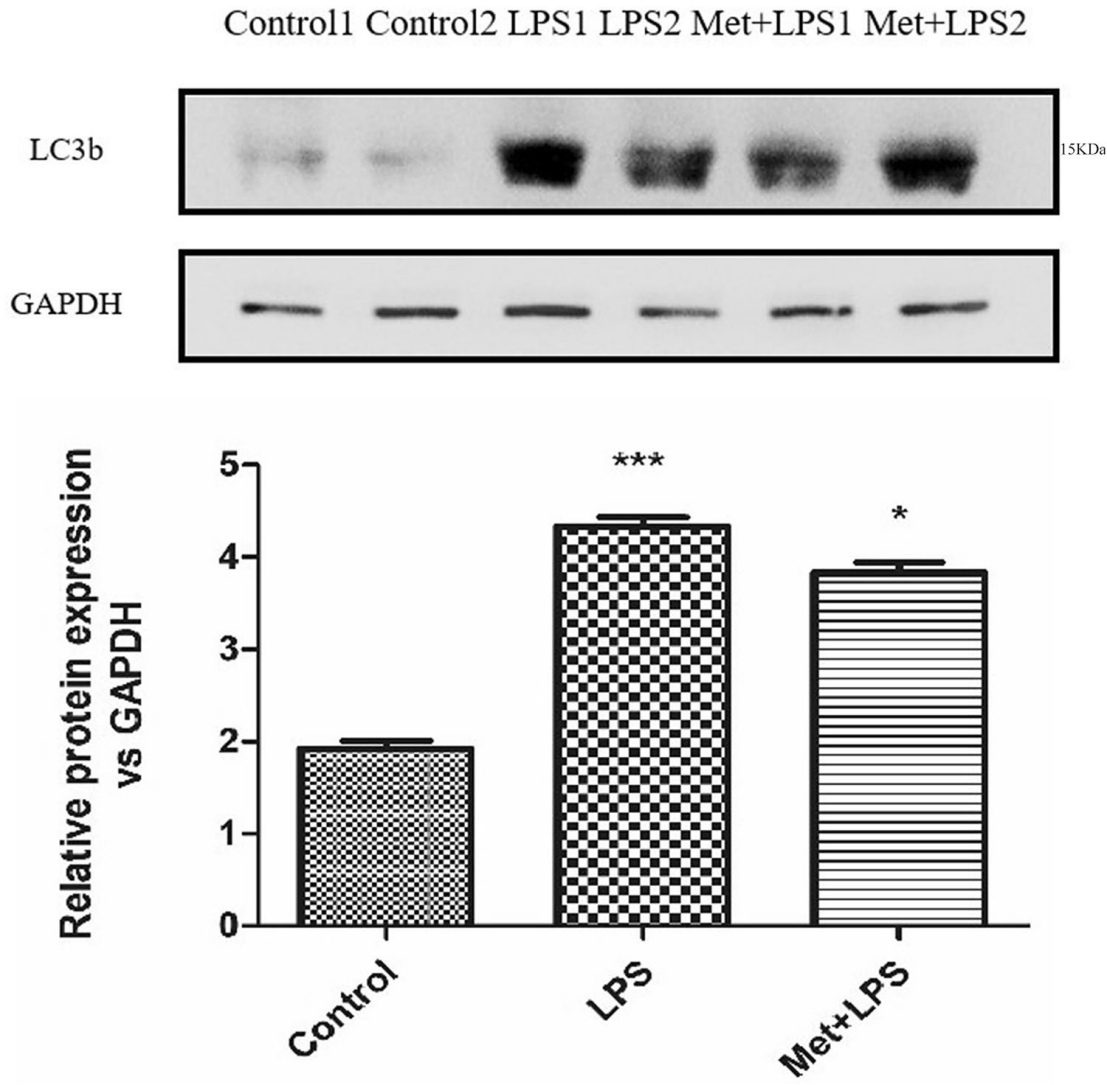
The expression levels of autophagy-related protein LC3b in macrophages in mice treated with lipopolysaccharide and metformin. GAPDH: glyceraldehyde-3-phosphate dehydrogenase, LPS: lipopolysaccharide, Met: Metformin, LC3b: microtubule associated protein 1 light chain 3 beta. LPS group: compared with blank control group. Met + LPS group: compared with LPS group. *P < 0.05, **P < 0.01, ***P < 0.0001.

We observed a significant increase of LC3b expression in the lung tissue of mice injected with LPS (*P* < 0.0001). However, this increase was reversed upon Met pre-treatment (*P* < 0.05) (Fig. 9).

## Discussion

In this study, we analyzed the expression of four enzymes involved in the TCA cycle upon LPS stimulation alone (inflammatory stimulation) and in the presence of Met (anti-inflammatory stimulation). Results showed that, in vitro*,* LPS induced the decrease of CS, OGDH, and IDH2, both at the mRNA and protein levels, which reverted in presence of Met. Similar results were obtained in vivo in a mouse model: upon LPS, the expression of OGDH, CS, and MDH2 in lung tissue decreased and the expression of OGDH and MDH2 was reverted upon Met pre-treatment.

A recent study described the role of IDH2 in cancer development^22^, while another suggested that the dysregulation of IDH2 in various cancers is correlated with inflammatory response disorders, such as the excessive production of pro-inflammatory factors^23^. In septic patients, the maximum level of CS activity in monocytes is significantly lower than that observed in normal people^24^. Thus, it has been suggested that a decrease in the expression of key enzymes of the TCA cycle could lead to an increase of reactive oxygen species, resulting in mitochondrial dysfunction, which may be correlated to septicemia. In the liver, the inflammatory reaction intensifies the acetylation of mitochondrial proteins such as MDH2. When MDH2 is acetylated, it activates the malate aspartate shuttle activity to maintain glycolysis^25^. OGDH is a key pro-inflammatory metabolic intermediate: through the NF-κB pathway, OGDH can increase the production of LPS-induced pro-inflammatory factors. The expression of OGDH is IDH2-dependent. When IDH2 is lost, the expression of OGDH decreases, reducing the LPS-induced pulmonary inflammatory response^26^.

Metabolites such as OGDH may be used as specific biomarkers of the human inflammatory response in the context of systemic infections, sepsis, and autoimmune diseases. The accurate determination of citric acid cycle intermediates is commonly performed using mass spectrometry, which is a fast, sensitive, and reproducible method, easy to standardize for clinical application^27^. Itaconic acid is a TCA cycle metabolite, which is believed to be released by macrophages during inflammation^28^. Succinic acid and citric acid have also been shown to be significantly reduced in the urine of patients affected with inflammatory bowel disease, compared to those observed in healthy people^29^. These limited clinical studies suggest that TCA cycle intermediates such as succinic acid, citric acid, itaconic acid, and fumaric acid may be easily measured in the body fluids of patients with different inflammatory conditions as biomarkers of the inflammation status^30^.

Our results also showed that, upon LPS treatment, ROS and autophagy increased, while the mitochondrial membrane potential decreased. These results suggest that inflammation would trigger autophagy to clear organelles damaged by ROS. Moreover, the decrease in the mitochondrial membrane potential suggests that mitochondrial function is negatively affected. We speculate that the expression of key enzymes and the efficiency of the TCA cycle decrease during the inflammatory state, leading to the accumulation of TCA cycle metabolites such as citric acid and succinic acid. The accumulation of these metabolites increases ROS levels. Increased ROS levels may inhibit the oxidative phosphorylation process and ATP formation, resulting in a damaged TCA cycle imbalanced mitochondrial energy metabolism, and a drop of membrane potential. In these conditions, autophagy can maintain cell homeostasis and protect the body from the inflammatory response triggered by ROS. Therefore, we hypothesize that inflammation in macrophages disrupts the TCA cycle, affecting the mitochondrial and cellular functions.

In this study, we used Met as an anti-inflammatory agent. Met is the first-line drug for the treatment of type 2 diabetes mellitus, which has been known for its anti-inflammatory effects^9,31^. Met can reduce the production of different pro-inflammatory factors and induce anti-inflammatory factors, regulating the differentiation of immune cells^32^. Previous studies have shown that Met could be a promising therapeutic agent for treating heat damage, as it significantly improved the mitochondrial energy in elderly mice^33^. Our results suggest that Met can protect cells from the inflammation-induced dysregulation of the TCA cycle. Therefore, we speculate that the mechanism underlying the use of Met to treat heat injury may be correlated to its ability to protect mitochondrial activity. Considering the great potential of Met for the treatment of different diseases^34–43^, further investigation of its anti-inflammatory potential and its role in energy metabolism and mitochondria regulation are needed.

Our study has some limitations as following: (1) the absence of the experimental group with alone metformin affects the interpretability of the results; (2) GAPDH as an internal reference in this study may increase the uncertainty of the results; (3) metformin administered to the animal model might induce an acute hypoglycemia episode that may affect the results; however, Met plus LPS group in this study did not occur an hypoglycemia episode, and its mechanism may be related to insulin resistance and hyperglycemia induced by LPS injection^44^; and (4) unfortunately, we have not been able to test the activities of the TCA cycle enzymes from other markers of oxidative stress and autophagy. Therefore, a validation study that investigates the molecular mechanisms regulating the TCA cycle upon inflammation stimulation should continue in the future.

## Supplementary information

Supplementary information.

## Data Availability

The data used to support the findings of this study are available from the corresponding author upon request.
