# Supplementary material for: Effects of inflammatory and anti-inflammatory environments on the macrophage mitochondrial function
Source: Sci Rep. 2020 Nov 23;10:20324. doi: 10.1038/s41598-020-77370-x (PMC7684315; doi:10.1038/s41598-020-77370-x)
Supplement: Supplementary file 1 — Supplementary information. [file 41598_2020_77370_MOESM1_ESM.pdf]

Effects of inflammatory and anti-inflammatory environments on the macrophage mitochondrial function

Dong Ji[1#], Jian-yun Yin[2#], Dan-feng Li[1], Chang-tai Zhu[1*], Jian-ping Ye[1*], Yuan-qing Pan[3*]

[1] Central Laboratory, Shanghai Jiao Tong University Affiliated Sixth People's Hospital, Shanghai 200233, China

[2] Department of Thyroid Breast Surgery, Kunshan Hospital Affiliated to Nanjing University of Traditional Chinese Medicine, Kunshan 215300, China

[3] Department of Medical Psychology, Guilin Medical College, Guilin 541010, China

[#]Dong Ji and Jian-yun Yin contributed equally to the study.

Competing interests

The authors declare no competing interests.

Source of financial support

The work was supported by the Natural Science Foundation of China (NO. 81372212).

Corresponding authors:

Chang-tai Zhu Shanghai Jiao Tong University Affiliated Sixth People's Hospital No. 600 Yishan Road, Shanghai 200233, China. Tel: +86-021-38297710. Email: zct101@163.com

Jian-ping Ye Shanghai Jiao Tong University Affiliated Sixth People's Hospital No. 600 Yishan Road, Shanghai 200233, China. Tel: +86-021-38297716. Email:yejianping@sjtu.edu.cn

Yuan-qing Pan Department of Medical Psychology, Guilin Medical University, No.1 Zhiyuan Road, Guilin 541010, China. Tel: +086-0773-3661865. Email: panyuanq@126.com

EMAILS

Dong Ji

903161136@qq.com

Jian-yun Yin

yinjianyun99@sina.com

Dan-feng Li
ldfyeah@163.com

Chang-tai Zhu
zct101@163.com

Jian-ping Ye
yejianping@sjtu.edu.cn

Yuan-qing pan
panyuanq@126.com

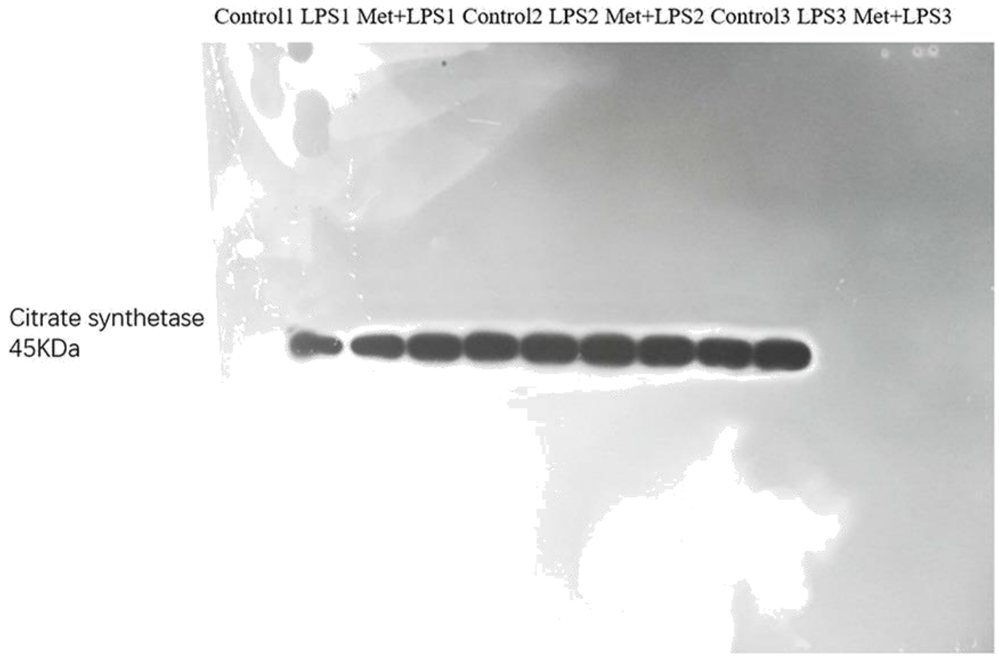

S1 Expression levels of key enzymes in mitochondrial tricarboxylic acid cycle of macrophages under the stimulation of inflammatory and anti-inflammatory agents
LPS: lipopolysaccharide, Met: Metformin, CS: citrate synthase

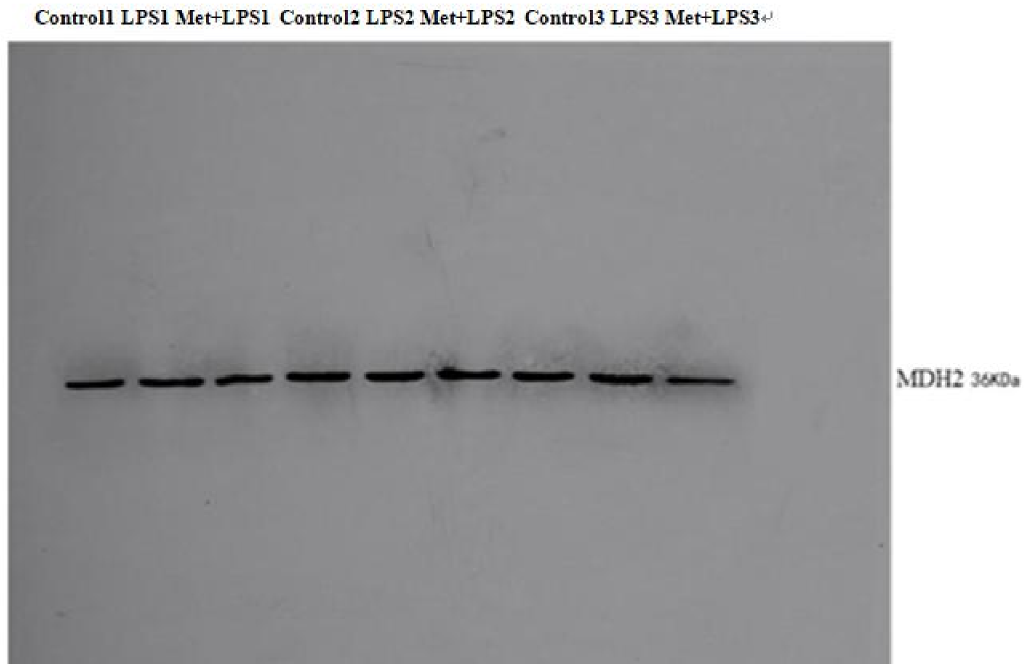

S2 Expression levels of key enzymes in mitochondrial tricarboxylic acid cycle of macrophages under the stimulation of inflammatory and anti-inflammatory agents
LPS: lipopolysaccharide, Met: Metformin, MDH: malic dehydrogenase

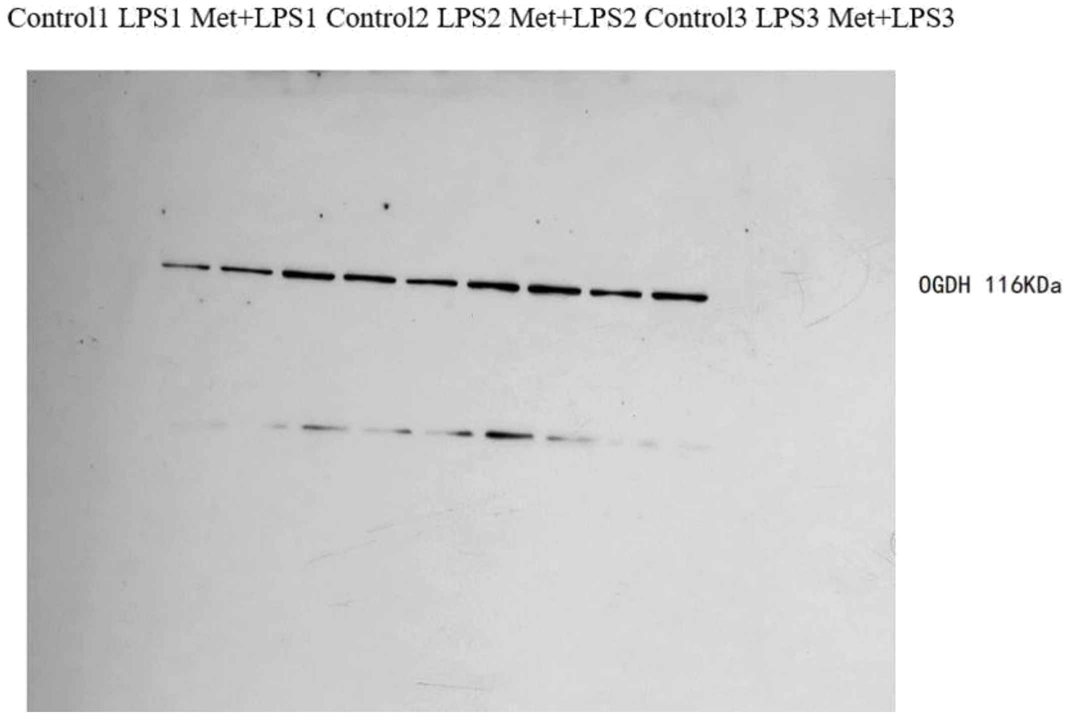

S3 Expression levels of key enzymes in mitochondrial tricarboxylic acid cycle of macrophages under the stimulation of inflammatory and anti-inflammatory agents

LPS: lipopolysaccharide, Met: Metformin, OGDH: α-ketoglutarate dehydrogenase

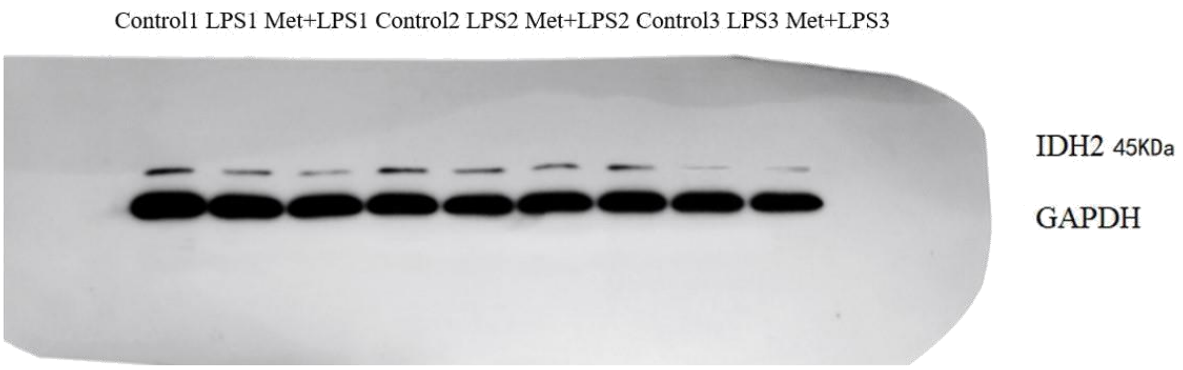

S4 Expression levels of key enzymes in mitochondrial tricarboxylic acid cycle of macrophages under the stimulation of inflammatory and anti-inflammatory agents GAPDH: glyceraldehyde-3-phosphate dehydrogenase, LPS: lipopolysaccharide, Met: Metformin, IDH: isocitrate dehydrogenase

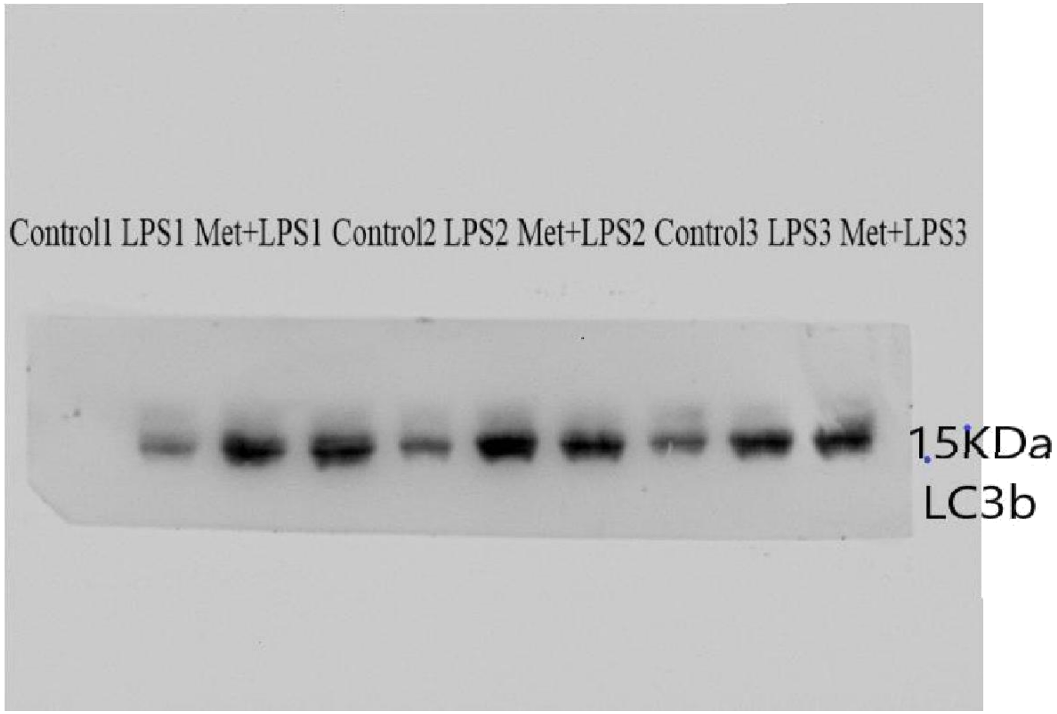

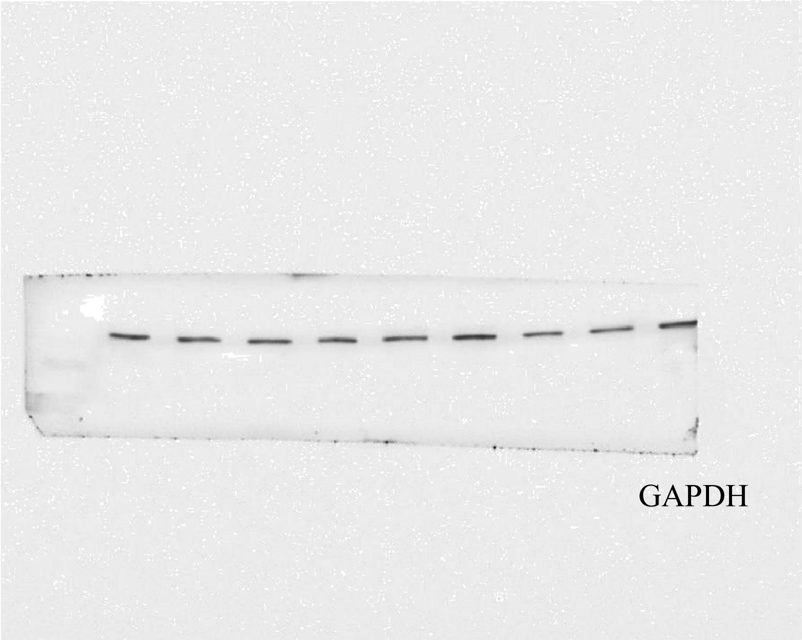

S5 Expression levels of key enzymes in mitochondrial tricarboxylic acid cycle of macrophages under the stimulation of inflammatory and anti-inflammatory agents GAPDH: glyceraldehyde-3-phosphate dehydrogenase, LPS: lipopolysaccharide, Met: Metformin, LC3b: microtubule associated protein 1 light chain 3 beta

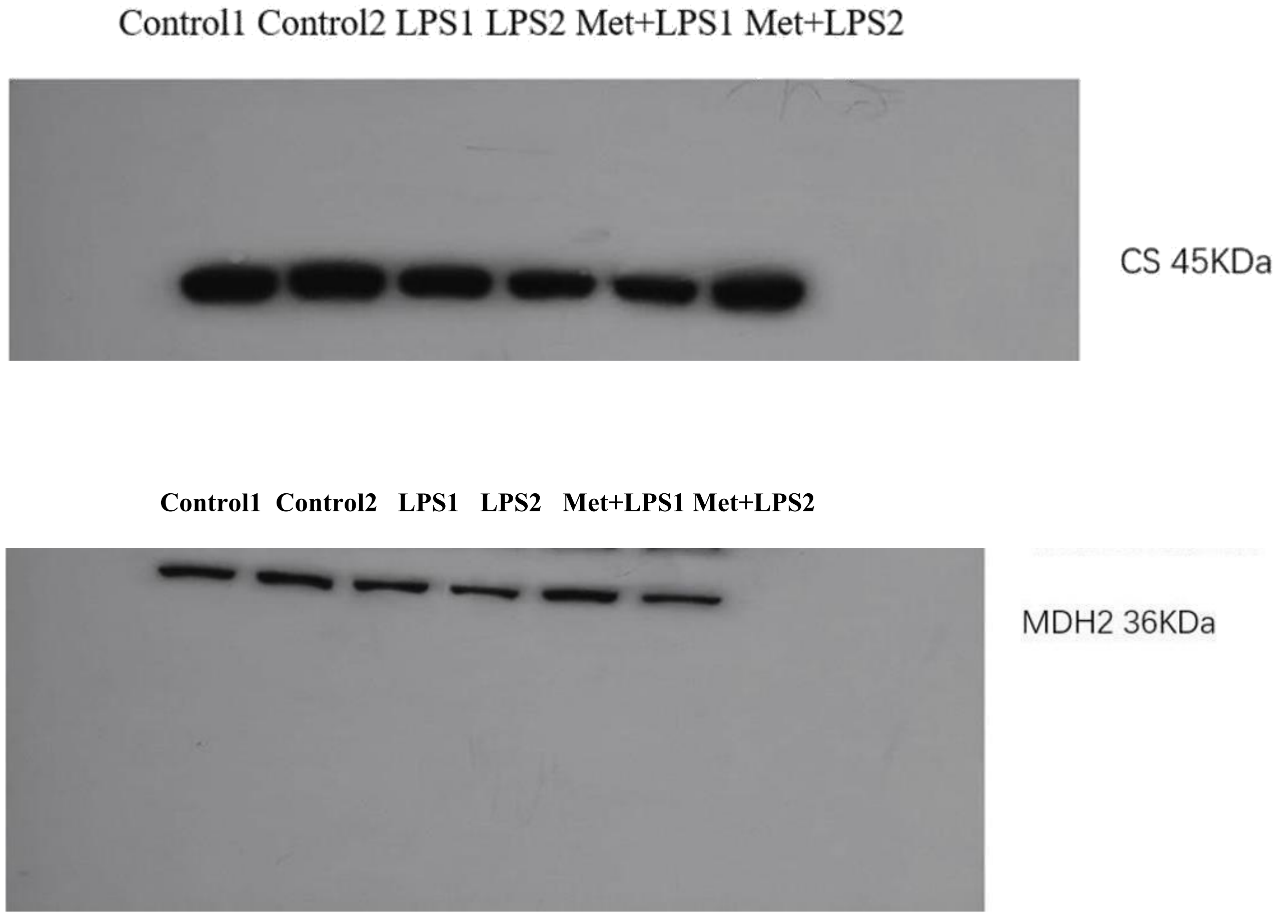

S6 Expression levels of key enzymes in mitochondrial tricarboxylic acid cycle of septic mice under the stimulation of inflammatory and anti-inflammatory agents

LPS: lipopolysaccharide, Met: Metformin, CS: citrate synthase, MDH: malic dehydrogenase

Control1 Control2 LPS1 LPS2 Met+LPS1 Met+LPS2

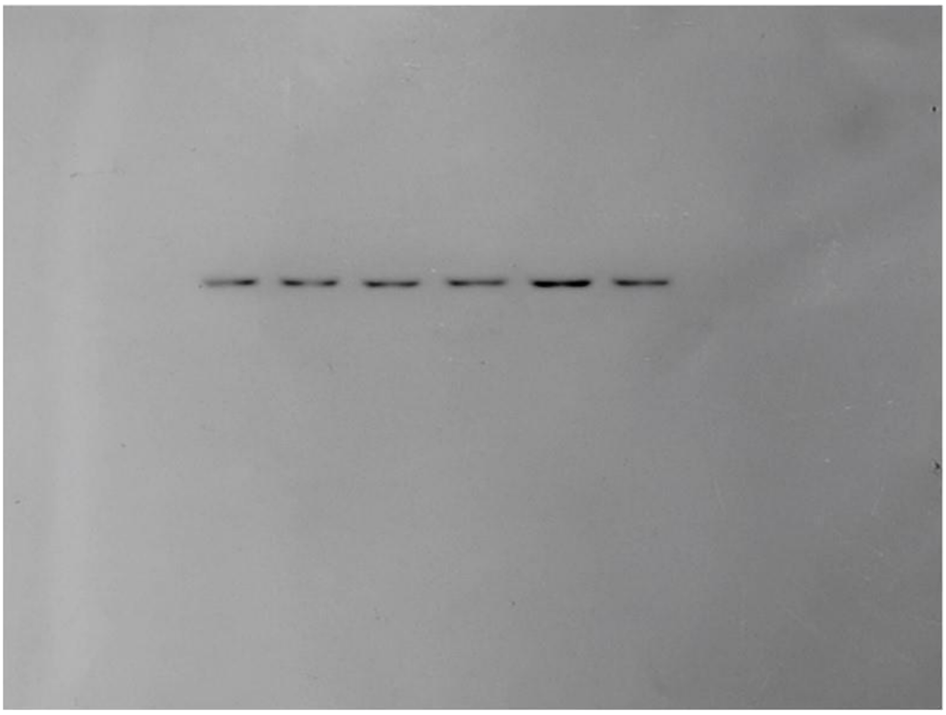

IDH2 45KDa

S7 Expression levels of key enzymes in mitochondrial tricarboxylic acid cycle of septic mice under the stimulation of inflammatory and anti-inflammatory agents

LPS: lipopolysaccharide, Met: Metformin, IDH: isocitrate dehydrogenase

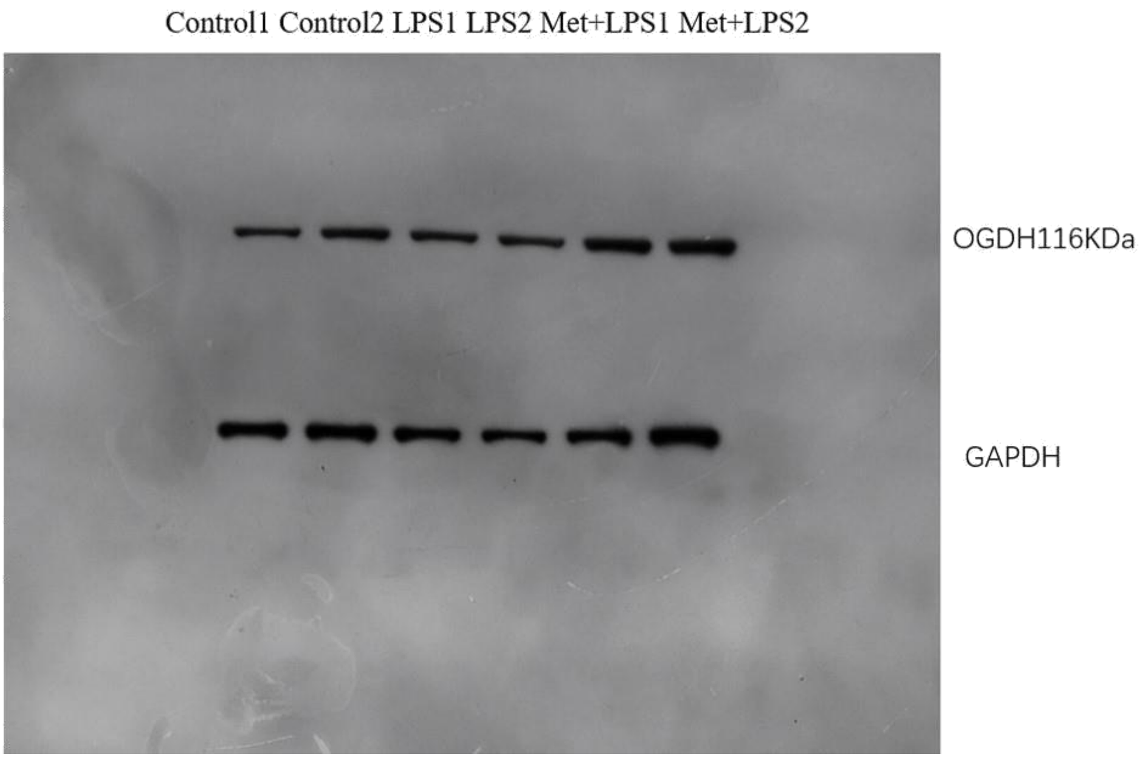

S8 Expression levels of key enzymes in mitochondrial tricarboxylic acid cycle of septic mice under the stimulation of inflammatory and anti-inflammatory agents.

LPS: lipopolysaccharide, Met: Metformin, OGDH: α -ketoglutarate dehydrogenase，GAPDH: glyceraldehyde-3-phosphate dehydrogenase.

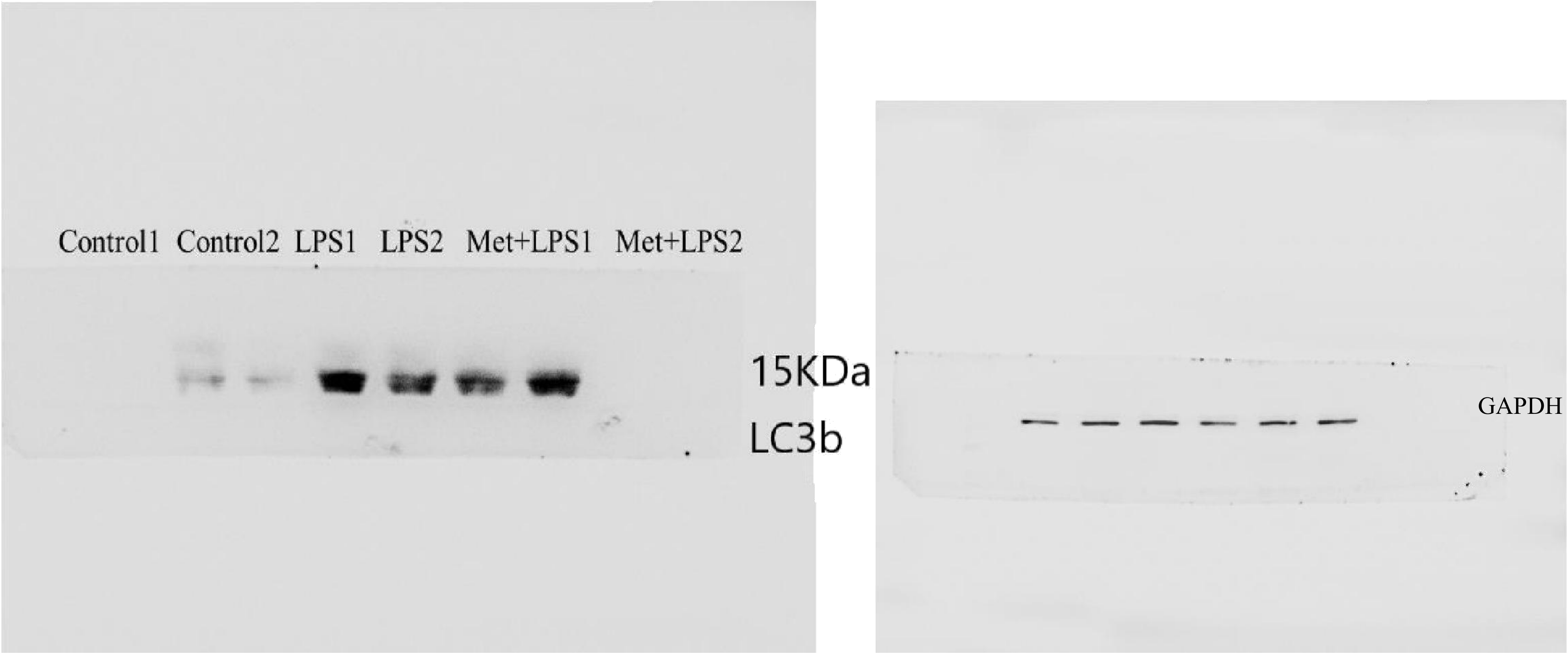

S9 Expression levels of key enzymes in mitochondrial tricarboxylic acid cycle of septic mice under the stimulation of inflammatory and anti-inflammatory agents LPS: lipopolysaccharide, Met: Metformin, GAPDH: glyceraldehyde-3-phosphate dehydrogenase, LC3b: microtubule associated protein 1 light chain 3 beta
